# P-1854. Children with Congenital Heart Disease and PICS (Persistent Inflammation, Immunosuppression, and Catabolism Syndrome) Demonstrate Both Pro- and Anti-Inflammatory Cytokine Profiles

**DOI:** 10.1093/ofid/ofae631.2015

**Published:** 2025-01-29

**Authors:** Natalya M Beneschott, Sandy M Yoder, Eric Brady, Amanda K Williams, C Buddy Creech

**Affiliations:** Vanderbilt University Medical Center, Nashville, Tennessee; Vanderbilt University Medical Center, Nashville, Tennessee; Vanderbilt University Medical Center, Nashville, Tennessee; Vanderbilt University Medical Center, Nashville, Tennessee; Vanderbilt University Medical Center, Nashville, Tennessee

## Abstract

**Background:**

Patients with chronic critical illness have variable recovery from acute events, such as surgery or sepsis, and experience dysregulated inflammation, blunted immune responses, and recurrent infections. The development of persistent inflammation, immunosuppression, and catabolism syndrome (PICS) increases the risk of healthcare associated infections, poor wound healing, and indolent organ failure. The pathophysiology of PICS has not been characterized in critically ill pediatric populations, particularly those with congenital heart disease (CHD).

**Figure:**
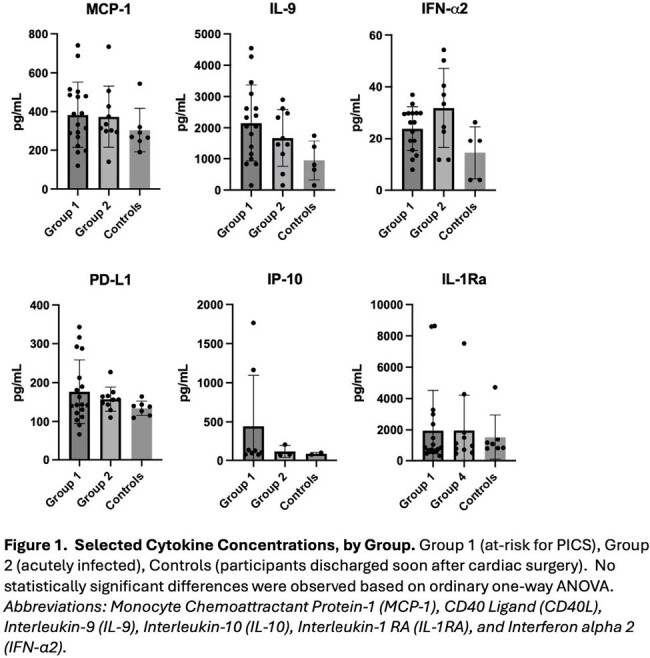

**Methods:**

The PICASSO study is an ongoing, observational cohort study of immunologic dysregulation in children with CHD who undergo cardiopulmonary bypass for surgical repair. Three groups were defined in this preliminary analysis. Children with uncomplicated post-operative courses served as control participants. Group 1 includes children who underwent CHD repair and were anticipated to have prolonged recovery (n=6), and Group 2 includes children with culture-confirmed, post-operative infection (n=2). PICS was defined as having at least 2 of the following criteria: lymphocyte count < 1500 cells/mL, CRP > 10 mg/dL, and albumin < 3 mg/dL. Blood samples were collected weekly for up to one month in Groups 1 and 2; controls had one sample collected at discharge. Plasma cytokines were measured using a 25-plex panel (Human Immunotherapy Fixed Panel, Luminex®).

**Figure:**
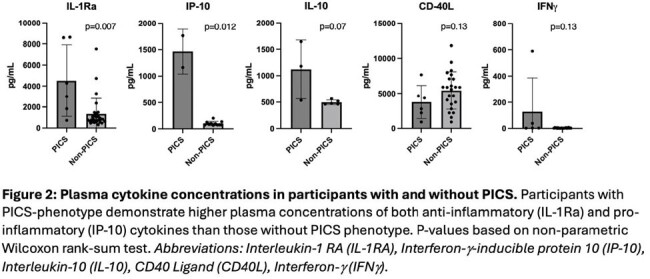

**Results:**

Thirty-four samples from 15 patients were analyzed. Plasma cytokines analysis across groups is shown in Figure 1. While come cytokines were more abundant in Group 1 compared to Group 2 and controls (e.g., IL-9 and IP-10), no significant differences were observed. Next, we compared cytokine levels in participants with (n=4) and without PICS (n=11). Both IL-1Ra and IP-10 were increased among children with PICS (p=0.007 and p=0.012, respectively).

**Conclusion:**

In a preliminary analysis of the first 15 patients in the cohort, children with CHD and PICS demonstrate increased plasma levels of both pro-inflammatory (IP-10) and anti-inflammatory (IL-1Ra) cytokines that mirror the clinical phenotype of PICS. Ongoing analysis includes immunophenotyping and immunometabolic profiling of peripheral blood mononuclear cells.

**Disclosures:**

**C. Buddy Creech, MD, MPH**, CommenseBio: Advisor/Consultant|GSK: Advisor/Consultant|Moderna: Advisor/Consultant|Moderna: Grant/Research Support|Sanofi Pasteur: Advisor/Consultant

